# Objective and Subjective Appetite Assessment in Patients with Gynecological Cancer: A Pre- and Post-Operative Pilot Study

**DOI:** 10.3390/ijerph191610322

**Published:** 2022-08-19

**Authors:** Iro-Spyridoula Gounitsioti, Dimitrios Poulimeneas, Maria G. Grammatikopoulou, Charalambos Kotzamanidis, Konstantinos Gkiouras, Meletios P. Nigdelis, Dimitrios Tsolakidis, Alexios Papanikolaou, Basil C. Tarlatzis, Dimitrios P. Bogdanos, Maria Tsigga, Dimitrios G. Goulis

**Affiliations:** 1 Department of Nutritional Sciences & Dietetics, Alexander Campus, International Hellenic University, Sindos, GR-57400 Thessaloniki, Greece; 2 Department of Nutrition and Dietetics, Harokopio University, E. Venizelou 70, GR-17671 Athens, Greece; 3 Department of Rheumatology and Clinical Immunology, University General Hospital of Larissa, Faculty of Medicine, School of Health Sciences, University of Thessaly, Biopolis, GR-41110 Larissa, Greece; 4 Hellenic Agricultural Organization-DEMETER, Veterinary Research Institute of Thessaloniki, Thermi Campus, GR-57001 Thermi, Greece; 5 Unit of Reproductive Endocrinology, 1st Department of Obstetrics and Gynecology, Medical School, Faculty of Health Sciences, Aristotle University of Thessaloniki, GR-56429 Thessaloniki, Greece; 6 1st Department of Obstetrics and Gynecology, Medical School, Faculty of Health Sciences, Aristotle University of Thessaloniki, GR-56429 Thessaloniki, Greece; 7 Second Department of Obstetrics and Gynaecology, Hippokration General Hospital, 49 Konstantinoupoleos Str., Aristotle University of Thessaloniki, GR-54642 Thessaloniki, Greece

**Keywords:** ovarian cancer, endometrial cancer, vaginal cancer, gynecological malignancy, nutritional status, anorexia, nutritional screening, cancer cachexia, sarcopenia, GLIM, SGA

## Abstract

Although appetite and its disorders have been implicated in disease progression and outcomes, ghrelin concentrations, an objective appetite measure, are rarely assessed in patients with gynecological malignancies. The present study aimed to assess changes in post-operative versus pre-operative appetite levels in patients with gynecological cancers scheduled for tumor removal surgery (N = 53). Acylated ghrelin concentrations were assessed as an objective appetite proxy, whereas the Council of Nutrition appetite questionnaire (CNAQ) was employed as a subjective appetite measure. Ghrelin concentrations were increased post-operatively (median: 12.1 pg/mL, IQR: 0.67 to 23.5, *p*-value = 0.001) but the perceived appetite of patients (CNAQ) remained unchanged (median: −1, IQR: −3 to 1). Tumor removal surgery decreased all anthropometric indices (body weight, body mass index, waist and hips circumferences, triceps skinfolds, body fat, fat mass and fat mass index, *p*-value ≤ 0.001 for all) and doubled the risk of malnutrition among patients. No difference was recorded in the change in participants’ objective and subjective appetite when they were classified according to the tumor type. No correlation was observed between ghrelin concentrations and CNAQ score pre-operatively (Spearman’s rho correlation coefficient = −0.181, *p*-value = 0.298) or post-operatively (Spearman’s rho correlation coefficient = 0.071, *p*-value = 0.684). The observed post-operative rise in ghrelin concentrations is associated with body weight loss and consists of a possible defense mechanism of the human body, aiming to prolong survival.

## 1. Introduction

Gynecological cancers are prevalent, contributing to the global female morbidity and mortality rate [1]. Their diagnosis and treatment are often associated with a lower quality of life, disability and reduced sexual function [1,2,3], increased fatigue [4], greater risk of anxiety, depression [5], malnutrition [6,7,8] and appetite loss [9], often leading to the development of sarcopenia and cancer cachexia [10,11].

In particular, appetite loss is an important effector of weight loss and malnutrition, which, in turn, may affect many prognostic clinical outcomes, including the length of hospital stay (LOS) [7,12] and morbidity [13]. Among patients with gynecological cancer, the prevalence of malnutrition is high [8,14] depending on the screening tool used, the therapeutic regime (radiotherapy, chemotherapy), the functional assessment of each patient [15], the existence of underlying depression [16] and the medical nutrition therapy applied. The most commonly used screening tool to identify patients with gynecological cancer at risk of malnutrition is the patient-generated subjective global assessment (PG-SGA) [17,18,19], which entails many limitations, including the high sensitivity, low objectivity and low agreement to the Global Leadership Initiative on Malnutrition (GLIM) criteria [20].

Objective appetite assessment measures, such as appetite peptides (serum ghrelin, peptide YY, leptin), are considered predictors of cancer progression and metastasis, influencing disease outcomes, survival and malnutrition [21,22]; nevertheless, ghrelin concentrations are rarely evaluated in everyday clinical practice. On the other hand, subjective appetite assessment methods consist of faster and easier alternatives, often used by dietitians and medical practitioners to evaluate the degree of appetite loss [23] and to triage patients in need of nutrition interventions.

With this in mind, the present study aimed to assess changes in acute post-operative versus pre-operative appetite levels in patients with gynecological cancers scheduled for tumor removal surgery and the correlation between subjective and objective appetite assessment methods at each time point (pre- and post-operation).

## 2. Materials and Methods

### 2.1. Patient Recruitment

The sample included 53 gynecological oncology patients scheduled for removal operations. The recruitment occurred in the 1st Department of Obstetrics and Gynecology, situated at the Papageorgiou General Hospital, Aristotle University of Thessaloniki, in Greece, and sampling was performed during 2012–2013. Each case was staged according to the International Federation of Gynecology and Obstetrics (FIGO) classification [24].

Patient inclusion criteria involved (1) diagnosis of gynecological cancer, (2) scheduled for an operation, (3) communicating effectively in the Greek language, and (4) being willing to participate. Exclusion criteria for the study included (1) diagnosis of polycystic ovary syndrome (PCOS) or (2) any metabolic disorder that could affect ghrelin concentrations.

All data were collected at two time points, one day before the operation and one month after the operation. The characteristics of the sample are presented in Table 1.

Permission for the study was granted by the Papageorgiou Hospital’s Ethics Committee (157/27-06-2012). Patients were informed about the study’s nature and purpose and written informed consent was obtained before participation.

### 2.2. Anthropometry and Body Composition

Body weight was measured in the morning hours by an experienced dietitian (I.-S.G.) using a digital scale (SECA 813, SECA Group, Hamburg, Germany). Height was measured using a wall-mounted stadiometer (SECA 216, SECA Group, Hamburg, Germany). Body mass index (BMI) was calculated for each patient by dividing body weight (kg) by the square of height (m). Weight status was classified according to the World Health Organization (WHO) BMI thresholds [25].

Waist and hip circumferences were measured using a common non-elastic tape with subjects on a horizontal plane [26] following the WHO procedures.

Skinfold thickness was measured three times at the triceps, thigh and suprailiac sites using a set of Lange calipers (Creative Health Products, Ann Arbor, MI, USA). The median measurement at each body site was used for all analyses. For adult women, body fat was calculated as the percent of body weight, using the Siri equations [27] and the Jackson–Pollock–Ward formula [28]. Calculation of the fat mass index (FMI) and the fat-free mass index (FFMI) was performed for all participants as total fat mass (kg) divided by height (m^2^) and total fat-free mass (kg) divided by height (m^2^), respectively [29].

### 2.3. Malnutrition Assessment

Two approaches were used to identify malnourished women: the subjective global assessment (SGA) [30] and the GLIM [31] criteria.

The SGA was used to identify malnutrition according to Detsky [32]. The SGA is a widely used tool [30], validated for oncological patients, which assesses dietary intake, changes in body weight, symptoms, functional capacity, metabolic requirements and patients’ physical examination results. One experienced dietitian (I.-S.G.) and two gynecologists (D.T. and A.P.) examined each patient and classified participants into well-nourished (A), mildly/moderately (B) or severely malnourished (C) SGA categories [32].

The GLIM criteria were also applied to identify malnourished patients in a more contemporary and universal approach [31]. Malnutrition was diagnosed among women with at least one phenotypic (non-voluntary weight loss, low BMI and muscle mass) and one etiologic criterion (inflammation and disease burden, reduced food intake or assimilation). Concerning the phenotypic criteria, patients with a BMI < 20 kg/m^2^ were considered to have a low BMI, those with an FFMI < 15 kg/m^2^ were considered to have low muscle mass [33] and non-volitional weight loss was also recorded as in the SGA based on previous body weight records of the patients. With regard to the etiological criteria, disease burden inflammation was considered as a criterion fulfilled by all patients due to the known cancer diagnosis and reduced food intake, or assimilation was noted when <50% of the energy requirements were met, according to evaluation by a dietitian (I.-S.G.) based on the GLIM consensus [31].

### 2.4. Blood Sample Collection Ghrelin Assay and Biomarkers

Venous blood samples were collected from 35 participants in the early morning after an overnight fast. Due to the instability of ghrelin, standard collection methods were applied to increase the accuracy of the assay [34]. For each mL of blood, 50 μL of AEBSF [4-(2-Aminoethyl)-benzene sulfonyl fluoride] was added, and samples were transferred into Eppendorf tubes and stored at −20 °C until analysis. Blood samples were analyzed to determine acylated ghrelin, creatinine, glucose, total protein and albumin concentrations. Glucose, creatinine, albumin and total protein were analyzed using the relevant kits available from Abbot Laboratories through an automatized analyzer (ARCHITECT c3000, Abbott Laboratories, Abbott Park, IL, USA), according to the manufacturer’s instructions.

### 2.5. Objective Appetite Assessment (Acylated Ghrelin Assay)

Serum acylated ghrelin concentrations were assessed with a commercially available human acylated ghrelin enzyme-linked immunosorbent assay (ELISA) kit (Catalog # A05106, SPI-BIO, Human Acylated Ghrelin Enzyme Immunoassay Kit, France), according to manufacturer guidelines. The kit’s detection limits for acylated ghrelin was 1.5, and the intra- and inter-assay coefficients of variation were 6.2% and 6.7%, respectively. The samples were spectrophotometrically read at a wavelength of 414 nm with an ELISA device (Power Wave Microplate Spectrophotometer, BIOT-TEK Instruments, Inc., Winooski, VT, USA). Results were calculated in pg/mL by multiplying the values of the dilution factor.

### 2.6. Subjective Appetite Assessment (CNAQ)

The Council of Nutrition appetite questionnaire (CNAQ), an 8-item single-domain short test, was applied to assess patients’ subjective appetite [35]. Responses to each question receive a 5-point score on a Likert-type scale. The total CNAQ score is calculated by the sum of each question’s score, ranging from 8 (worst) to 40 (best), with low scores being indicative of appetite deterioration [35]. A total CNAQ score ≤ 28 may identify persons with anorexia at risk of significant (>5%) body weight loss.

### 2.7. Statistical Analyses

Normality was assessed visually and by the use of the Shapiro–Wilk’s test. Normally distributed variables are presented as mean ± standard deviation (SD), whereas non-normally distributed as median (interquartile range (IQR)). For convenience in the analyses, mildly/moderately malnourished (B) and severely malnourished (C) patients, according to the SGA, were grouped. The paired t-test, Wilcoxon signed-rank test and McNemar’s test were used to compare variables before and after the intervention. Spearman’s rho correlation coefficient was used to assess the correlation between ghrelin concentrations and CNAQ, pre- and post-operative values. Due to the relatively small number of participants, regression analyses could not be performed.

The level of significance (*p*-value) was set at 0.05 unless otherwise specified. The analyses were conducted using the Statistical Package for Social Sciences (SPSS) software version 25.0 (SPSS, Chicago, IL, USA) and the *R* language (1.2.5033).

## 3. Results

### 3.1. Post-Operative Changes in Anthropometric Indices and Malnutrition Diagnosis

Table 2 details changes in anthropometric and malnutrition diagnosis post-operatively compared with the pre-operative values. Body weight reduced by −2.3 ± 0.5 kg, reflecting a −3.0 ± 0.6% change from the sample’s baseline body weight. In general, all anthropometric indices of the sample were significantly reduced post-operatively, doubling the overall malnutrition prevalence.

The SGA was more sensitive in diagnosing malnutrition in the sample; although, both diagnostic tools (SGA and GLIM) revealed a double risk of malnutrition post-operatively (Table 2).

Furthermore, no correlation was observed between acylated ghrelin concentrations and CNAQ pre-operatively (Spearman’s rho correlation coefficient = −0.181, *p*-value = 0.298) or post-operatively (Spearman’s rho correlation coefficient = 0.071, *p*-value = 0.684).

### 3.2. Post-Operative Changes in Appetite and Biochemical Markers

Post-operative changes in objective and subjective appetite and selected biochemical markers are presented in Table 3. The removal operation induced an increase in circulating fasting glucose concentrations, which coincided with increased ghrelin concentrations. However, no differences were noted concerning the CNAQ score of participants.

### 3.3. Type of Surgery, Appetite Levels and Other Indices

Table 4 compares changes in objective (ghrelin concentrations) and subjective (CNAQ) appetite before and after removal surgery across patients with different gynecological malignancy sites. With only three women diagnosed with vulvar cancer, these comparisons were omitted from the analyses. Despite the different operations performed, no differences were noted in either serum ghrelin concentrations or the CNAQ score between patients with ovarian, endometrial or cervical malignancy.

## 4. Discussion

The present study revealed that removal operations for gynecological cancers impact anthropometric indices, multiplying patient malnutrition risk. In parallel, a rise in serum ghrelin concentrations was observed, although it was not accompanied by a respective change in the patients’ perceived appetite. No effect was noted on ghrelin concentrations or the CNAQ score among women who underwent operations for different malignancy types. The present study also verified differences between the SGA and the GLIM criteria concerning nutrition assessment of patients with gynecological cancer.

The conducted operations for tumor removal reduced all anthropometric indices and doubled the risk of malnutrition in the sample. According to Santarpia [36], the surgery may interact with malnutrition status by inducing changes in patients’ pharmacokinetics, metabolism and healing dynamics. Pre-operative malnutrition is an important effector of post-operative infections, complications, LOS in the ventilator and the intensive care unit and increased mortality [37].

Apart from a reduction in all anthropometric indices, the present study indicates that removal surgery for gynecological tumors is associated with a concomitant increase in ghrelin concentrations, although patients’ perceived appetite does not appear to change. Similarly, Hernández Morante et al. [38] reported that when moderate weight loss was induced among patients with obesity following a hypocaloric diet, ghrelin concentrations were increased, although the subjective appetite of participants, as assessed through an application (Dietavisa^®^), remained unaffected. In the same context, the Lifestyle, Exercise, and Nutrition (LEAN) trial revealed that greater weight loss was associated with increased ghrelin concentrations among female breast cancer survivors [39]. Irrespective of the cancer site, research indicates that weight loss associated with malignancies reduces circulating ghrelin concentrations [40]. Nevertheless, according to Sumithran [41], the hormonal mediators of appetite promoting weight gain remain relatively elevated, even one year after achieving weight loss, and might propel a weight gain relapse. As far as patients with cancer are concerned, even a single bout of chemotherapy is effective in reducing ghrelin concentrations [42], and this observation remains apparent even in the long term. Examining patients with esophageal cancer, Miyazaki et al. revealed that a reduction in BMI and plasma ghrelin concentrations persisted even at 6–24 months post-esophagectomy [43]. These observations indicate that the human body cannot distinguish between weight loss due to lack of food access or intentional weight loss and activates survival mechanisms like increased circulating ghrelin concentrations, in an effort to prevent starvation.

In the present sample, the observed post-operative rise in ghrelin concentration was accompanied by a subsequent elevation in fasting glucose levels. Circulating ghrelin and glucose concentrations are dovetailed, with rises in the former, subsequently increasing serum concentrations of the latter. In starvation or malnutrition cases, this synergy positively affects survival, protecting against life-threatening falls in blood glucose [44]. However, as with insulin, a resistance in ghrelin secretion has been reported among patients with obesity, resulting in lower ghrelin concentrations, while worsening glucose tolerance. According to Giammanco [45], these changes in hormone secretion rates are secondary to obesity, associated with hypothalamic inflammation [46] and might be reversed following a reduction in body weight. Nevertheless, exogenous ghrelin infusion has been shown to surpass this barrier by increasing blood glucose in humans of either weight status, with greater blood glucose increments occurring in particular among those with obesity [47]. In parallel, in vitro studies have suggested that ghrelin and D-Lys3-GHRP-6, an inhibitor of the ghrelin receptor to the ovarian cancer cells line HO-8910, induced the apoptosis of HO-8910 cells through phosphorylated ERK1/2 [48]. As a result, ghrelin has been proposed as a putative therapeutic option for cancer cachexia and/or malnutrition [49]. However, a recent Cochrane systematic review [50] pinpoints the limited available data and insufficient evidence regarding body weight changes post-ghrelin therapy.

A low correlation was noted between acylated ghrelin concentrations and CNAQ score, indicating that the latter is not as sensitive in depicting changes in appetite among patients with cancer. Compared with other subjective appetite measures, the CNAQ is more sensitive but has a lower specificity [51]. Nevertheless, health professionals using the questionnaire should be aware of its limited ability to detect appetite changes among patients with cancer, and whenever possible, a ghrelin assay should be performed.

Limitations of the present study include the relatively small number of participants, which did not allow for the performance of regression analyses. In parallel, not all participating women consented to the provision of blood samples for the assays. Furthermore, given the small number of patients undergoing chemotherapy/radiotherapy or being diagnosed with malnutrition using the GLIM criteria, we could not perform statistically sound subgroup analyses to further investigate our hypothesis. Assessment of other hormones involved in the regulation of hunger, including insulin and leptin, might have provided more information regarding the post-operative ghrelin reduction, yet these examinations could not be performed due to limited resources. Depression was not examined herein although it has been suggested to be an important driver of malnutrition in gynecological cancer [16]. Although a follow-up assessment of ghrelin, appetite and malnutrition at 3 or 6 months post-operatively might have provided more insight, this was not feasible as not all patients were seen by oncologists at the hospital where the operation took place, and as a result, follow-up assessments after 1 month post-operatively were not routinely scheduled for every patient in our clinic. Moreover, more follow-ups might increase the burden on the patients and lead to more dropouts. Nevertheless, this is the first study assessing the effect of tumor removal surgery on the appetite of patients with cancer in general, and in patients with gynecological cancer in particular, adding to the evidence in this field.

## 5. Conclusions

In conclusion, tumor removal surgery appears to multiply malnutrition risk among patients with gynecological cancer. In parallel, ghrelin concentrations are increased to combat this abrupt malnutrition threat, although patients’ perceived appetite remains unchanged. Research on the effects of tumor removal operations on appetite is lacking, and more studies are required to understand the underlying physiology and pathogenetic mechanisms.

## Figures and Tables

**Table 1:** Patient characteristics at baseline (*n*, or mean ± SD) (N = 53).

Age (years)	58.0 ± 14.0
Length of hospital stay (days)	10 ± 4
Site (ovarian/endometrial/cervical/vulvar) (*n*)	16/25/8/4
Classification * (I/Ia/Ib/Ib1/Ib2/III/IIIc/IV) (*n*)	1/17/6/4/2/2/3/12/8
Co-existing cancer (*n*)	8
Pre-existing cancer (*n*)	4
On chemotherapy/radiotherapy (*n*)	7/7
Parity	2.0 ± 0.9
Nulliparity (*n*)	3
Smokers (*n*)	16
Presence of ascites (*n*)	6
Menopausal women (*n*)	42
BMI (kg/m^2^)	30.0 ± 7.3
Weight status ^†^ (underweight/normal weight/overweight/obese) (*n*)	1/14/16/22

Results are given as absolute numbers or as mean ± standard deviation (SD). BMI, body mass index; SD, standard deviation; * according to the International Federation of Gynecology and Obstetrics system [24]; ^†^ according to the World Health Organization thresholds [25].

**Table 2:** Anthropometric characteristics of participants pre- and post-operatively (N = 53).

	Pre-Operatively	Post-Operatively	Significance
BW (kg)	75.1 ± 2.5	72.8 ± 2.5	0.001 ˆ
BMI (kg/m^2^)	30.0 ± 7.3	29.1 ± 7.3	0.001 ˆ
Waist circumference (cm)	102.7 ± 19.7	99.2 ± 19.8	0.001 ˆ
Hip circumference (cm)	108.6 ± 14.0	106.4 ± 15.0	0.001 ˆ
Triceps skinfold (mm)	25.9 ± 9.9	22.6 ± 8.8	0.001 ˆ
Body fat (%BW)	36.0 ± 7.7	27.3 ± 11.4	0.012 ˆ
Fat mass (kg)	30.1 ± 11.2	19.3 ± 9.3	0.001 ˆ
FMI (kg/m^2^)	10.1 ± 4.7	7.7 ± 3.8	0.001 ˆ
Malnutrition status ^†^ (SGA) (*n*, *%*)	9 (17%)	18 (34%)	0.013 ^‡^
Malnutrition status (GLIM) (*n*, *%*)	4 (7.5%)	8 (15.1%)	0.014 ^‡^

Results are provided as mean ± standard deviation (SD). BMI, body mass index; BW, body weight; FMI, fat mass index [29]; GLIM, Global Leadership Initiative on Malnutrition [31]; SGA, subjective global assessment [30]; ^†^ mildly/moderately malnourished (B) and severely malnourished (C) patients, grouped according to the SGA [32]; ˆ Paired *t*-test analyses; ^‡^ McNemar’s test.

**Table 3:** Post-operative changes in appetite and serum biochemical markers (*n* = 35).

	Pre-Operative (*n* = 35)	Post-Operative (*n* = 35)	Significance ^†^
Albumin (g/dL)	4.1 (2.4, 6.2)	4.7 (2.5, 4.1)	NS ^†^
Creatinine (mg/dL)	0.7 (0.5, 2.6)	0.7 (0.6, 75.0)	0.020 ^†^
Glucose (mg/dL)	86.0 (55.0, 132.0)	92.0 (63.0, 129.0)	0.015 ^†^
Total protein (g/L)	7.1 (3.8, 10.0)	7.3 (8.2, 0.8)	NS ^†^
Ghrelin (pg/mL) (*n* = 29)	50.1 (20.8, 242.9)	58.9 (21.5, 414.5)	0.001 ^†^
CNAQ score (*n* = 53)	30.5 ± 4.2	29.5 ± 4.2	NS ^†^
Low CNAQ score * (*n*) (*n* = 53)	10	15	NS ^‡^

Results are given as median (interquartile range [IQR]). CNAQ, Council of Nutrition appetite questionnaire [35]; NS, not significant; * indicative of low appetite; ^†^ Wilcoxon signed-rank test; ^‡^ McNemar’s test.

**Table 4:** Changes (Δ) in post-operative objective and subjective appetite assessment compared to the baseline, according to the site of gynecological cancer.

	Ovarian (*n* = 12)	Endometrial (*n* = 14)	Cervical (*n* = 6)	Significance ^†^
Δ Ghrelin (pg/mL)	−17.8 (−42.7 to 10.6)	−6.14 (−14.8 to −0.7)	−22.0 (−33.4 to −13.6)	NS
Δ CNAQ score	−0.5 (−3.0 to 0.3)	1.5 (0 to 3.8)	−0.5 (−1.8 to 0)	NS

Results are given as median (interquartile range [IQR]). CNAQ, Council of Nutrition appetite questionnaire [35]; NS, not significant; Δ, difference between baseline and post-operation values; ^†^ according to the Kruskal–Wallis test.

## Data Availability

Data will be provided upon request to the first two authors (I.-S.G and D.P.).
